# Differences in diagnostic activity in general practice and findings for individuals invited to the danish screening programme for colorectal cancer: a population-based cohort study

**DOI:** 10.1080/02813432.2018.1487378

**Published:** 2018-06-22

**Authors:** Jakob Søgaard Juul, Berit Andersen, Søren Laurberg, Anders Helles Carlsen, Frede Olesen, Peter Vedsted

**Affiliations:** a Research Unit for General Practice & Section for General Medical Practice, Department of Public Health, Aarhus University, Bartholins Allé 2, Aarhus C, 8000, Denmark;; b Research Centre for Cancer Diagnosis in Primary Care, Department of Public Health, Aarhus University, Bartholins Allé 2, Aarhus C, 8000, Denmark;; c Department of Public Health Programmes, Randers Regional Hospital, Skovlyvej 1, Randers, NE, 8930, Denmark;; d Department of Surgery, Aarhus University Hospital, Tage Hansens Gade 2, Aarhus C, 8000, Denmark;; e Department of Clinical Medicine, University Clinic for Innovative Patient Pathways, Silkeborg Hospital, Aarhus University, Denmark

**Keywords:** Cohort study, mass screening, colorectal neoplasm, Denmark, general practice

## Abstract

**Objective:** To investigate the diagnostic activity in general practice and the cumulative incidence of colorectal cancer (CRC) in individuals invited to the Danish national screening programme for CRC.

**Design:** A historical population-based cohort study.

**Setting:** The Danish CRC screening programme and general practice.

**Subjects:** The 376,198 individuals invited to the Danish CRC screening programme from 1 March to 31 December 2014.

**Main outcome Measures:** The diagnostic activity (consultations and haemoglobin measures) in general practice in the year preceding the screening invitation and the cumulated incidence of CRC in the year following the screening invitation.

**Results:** Screening participants had significantly higher diagnostic activity than non-participants. Individuals with a positive faecal immunochemical test (FIT) had higher diagnostic activity compared to individuals with a negative FIT, and a small increase in the months leading up to the invitation. Individuals with a screen-detected CRC had lower diagnostic activity than individuals with no CRC. In total, 308 (25.3%) of CRCs diagnosed in the invited population were diagnosed outside the screening programme. Non-participants with CRC more often had low socio-economic status, high comorbidity and stage IV CRC than participants with CRC.

**Conclusions:** There was a tendency that participants and those with a positive FIT had a higher diagnostic activity the year before the screening. This was not seen for those with CRC detected through screening. CRC must still be diagnosed in general practice in the invited population and non-participants are of special interest as they have higher risk of late stage CRC.

**Key Points**
- Current awareness:Individuals with colorectal cancer (CRC) in screening may be symptomatic and CRC may still occur outside screening in the invited population.
- Most important points:The majority of individuals with CRC in screening cannot be expected to be diagnosed on symptomatic presentation in general practice
- GPs have to be aware that CRC still occurs outside screening in the invited population
- Non-participants with CRC are often deprived and have late stage CRC

## Introduction

Colorectal cancer (CRC) is the third most common cancer worldwide and one of the main reasons for cancer-related death [1]. During decades, Denmark has had poorer CRC survival than other Nordic countries. This may partly be explained by late stage of disease at diagnosis [2]. In an attempt to identify cancer at earlier stages and thereby improve the CRC prognosis, a screening programme based on the use of the faecal immunochemical test (FIT) was implemented in March 2014 for individuals aged 50-74 years [3].

For individuals participating in the screening, recent questionnaire studies have indicated that lower gastrointestinal (GI) symptoms are prevalent in up to 70-80% of the individuals with a positive faecal test [4–6]. If this is true, these individuals may be detected before entering the screening programme through symptomatic presentation in general practice if the general practitioner (GP) had access to the FIT and thus, providing the opportunity to detect the CRC earlier.

For individuals invited to, but not participating in the screening, studies indicate that general practice will play a key role in the detection of CRC [7]. However, so far, no study has investigated the amount of CRCs occurring outside the Danish screening programme in the invited population, but for GPs this is of special interest since non-participants diagnosed with CRC may have low socio-economic status (SES) and poor CRC prognosis [8–10].

Thus, the aim of this study was to investigate the diagnostic activity in general practice and the cumulative incidence of CRC in individuals invited to the Danish national screening programme for CRC.

## Material and methods

### Study design

We conducted a historical population-based cohort study using registry data to assess the diagnostic activity in general practice and the cumulative incidence of CRC.

### Setting

From March 2014, CRC screening is being implemented over a 4-year period in Denmark and will be offered biennially after full implementation. The screening is free of charge and uses the FIT as a first line test, which is followed by colonoscopy (or alternatively CT-colonography) if the FIT is positive (≥100μg/L).

All citizens aged 50-74 years are invited to participate in the screening. Citizens participate by performing a FIT received in an invitational letter. The order in which citizens are invited is random and determined on the basis of their month of birth. However, citizens turning 50 or 74 years during the prevalence screening must receive a screening invitation before that particular birthday.

General practice is not involved in any part of the Danish screening programme for CRC. In Denmark, access to general practice is free of charge at the point of care and almost 99% of Danish citizens are listed with a general practice [11].

### Study population

All individuals invited to screening for CRC from 1 March to 31 December 2014 were eligible for inclusion. To ensure complete follow-up, we excluded individuals who were not listed with a GP, had lived outside Denmark at some point during the year preceding the screening invitation or had died within one month after receiving the invitation. Furthermore, to ensure a homogeneous population, we restricted the analyses to individuals without a previous diagnosis of colorectal disease (CRC, Crohn’s disease, ulcerative colitis, familial adenomatous polyposis (FAP), hereditary nonpolyposis colorectal cancer (HNPCC) or adenomas followed by regular colonoscopy).

### Outcome measures

*Diagnostic activity in general practice:* Daytime face-to-face consultations (incl. home visits) and point-of-care haemoglobin measurements in general practice (photometric analysis) were used as proxies for diagnostic activity in general practice related to gastrointestinal symptoms and signs, and assessed in the year preceding the screening invitation. The outcome was compared for participants vs. non-participants, individuals with positive FIT vs. individuals with negative FIT and CRC cases vs. non-CRC cases.

*Cumulative incidence of CRC:* Number of CRC diagnoses among individuals invited to screening in the year following the screening invitation. The CRC incidence was stratified into three subgroups: participants diagnosed in the screening, participants diagnosed outside the screening and non-participants.

### Data collection

Data on diagnostic activity was collected from the Danish National Health Service Register until three years preceding the screening invitation to allow for extension of the study period if differences were present for more than one year [12]. The database holds no information on results of laboratory analyses.

Data on CRC (ICD-10: DC180-9 and DC200-9) and UICC stages was collected from the Danish Cancer Registry from the day of invitation until one year following the screening invitation [13,14].

The Danish Colorectal Cancer Screening Database (DCCSD) provided information on the screening invitation, FIT results, performed colonoscopy or CT-colonography and screen-detected CRCs [15].

The Danish National Patient Register (NPR) was used to identify previous diagnoses of colorectal disease (Crohn’s disease (ICD-10: DK500-9), ulcerative colitis (ICD-10: DK510-9), FAP (ICD-10: DD126F), HNPCC (ICD-10: DC188A) and adenomas followed by regular colonoscopy (ICD-10: DZ018B) [16]. Furthermore, the NPR was used to identify diagnoses for generating the Charlson Comorbidity Index, which was categorised into “low” (CCI score = 0), “moderate” (CCI score = 1-2) and “severe” (CCI score = ≥3) (CCI) [17,18].

The Danish National Prescription Registry was used to collect information on prescriptions for medications against haemorrhoids (ATC: C05A) or drugs with anticoagulatory effect (NSAIDs (ATC: M01A), acetylsalicylic acids (ATC: B01AC06, N02BA01, N02BA51) and anticoagulants (ATC: B01A)) as these covariates were considered potential confounders for the outcomes [19].

Statistics Denmark provided data on vital status, socio-economic characteristics and demographic factors [20]. Marital status was dichotomised into living with a partner (“married/cohabitating”) or living alone (“alone”). Country of origin was categorised into "Danish”, “immigrant from a western country” or “immigrant from a non-western country”. Labour market affiliation was categorised into “working”, “unemployed” or “retirement pension”. Educational level was categorised into “basic” (<10 years), “medium” (10-15 years) and “high” (>15 years) [21].

All data were linked by the civil registration number [22].

### Statistical analysis

The index date was defined as the date the individual was invited to screening for CRC.

The diagnostic activity in general practice was investigated in the year preceding the screening invitation by estimating the consultation rates and haemoglobin measurements rates. This was done in intervals of three months. The diagnostic activity was compared between subgroups by estimating incidence rate ratios (IRRs) using negative binomial regression models with cluster robust variance estimation to account for heterogeneity between individuals. Estimates of the IRRs were adjusted for age, gender, marital status, country of origin, level of education, labour market affiliation, comorbidity, prescriptions of medicine against haemorrhoids and prescriptions of medicine with an anticoagulatory effect.

The cumulative incidence of CRC among individuals invited to screening was investigated in three subgroups to allow assessment of the number of CRCs diagnosed outside the screening programme. CRCs diagnosed among participants outside the screening programme were defined as; a CRC diagnosis after a negative FIT in the screening, or a CRC diagnosis registered in the DCR for an individual with a positive FIT in the screening, but no registered CRC diagnosis in the DCCSD.

In addition, we compared participants’ and non-participants’ risk of being diagnosed with a stage IV CRC by using a Poisson regression model with time at risk as exposure while taking into account the competing risk of getting a lower stage cancer.

All analyses were performed on the server of Statistics Denmark using Stata 14.0.

### Ethical approvals

The study was approved by the Danish Data Protection Agency (j.no. 2014-41-3143).

## Results

### Diagnostic activity in the year preceding the screening invitation

**Figure 1.**
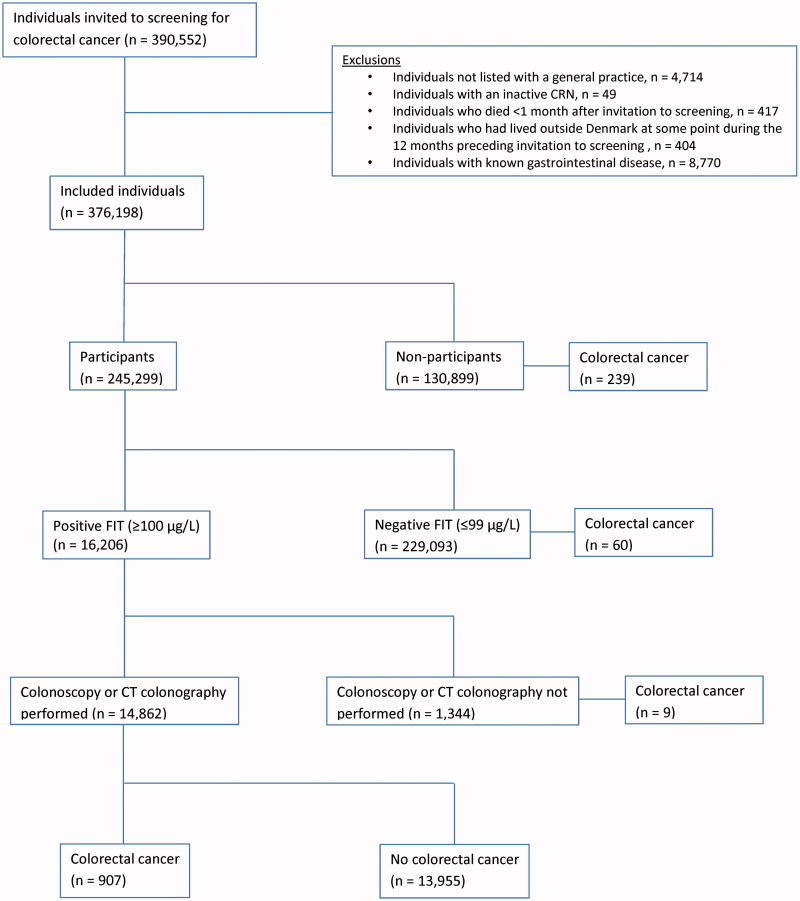
Flowchart of the study population. The number of CRC diagnoses for each subgroup is stated for the year following the screening invitation.

In total, 390,552 individuals were invited to screening from 1 March 2014 to 31 December 2014. After exclusions, 376,198 individuals were included in the analyses (Figure 1). Among the 245,299 (65%) participants, 16,206 (6.6%) had a positive FIT result and 907 (6.1%) of individuals who had a colonoscopy or CT-colonography performed were diagnosed with CRC (Figure 1).

**Figure 2.**
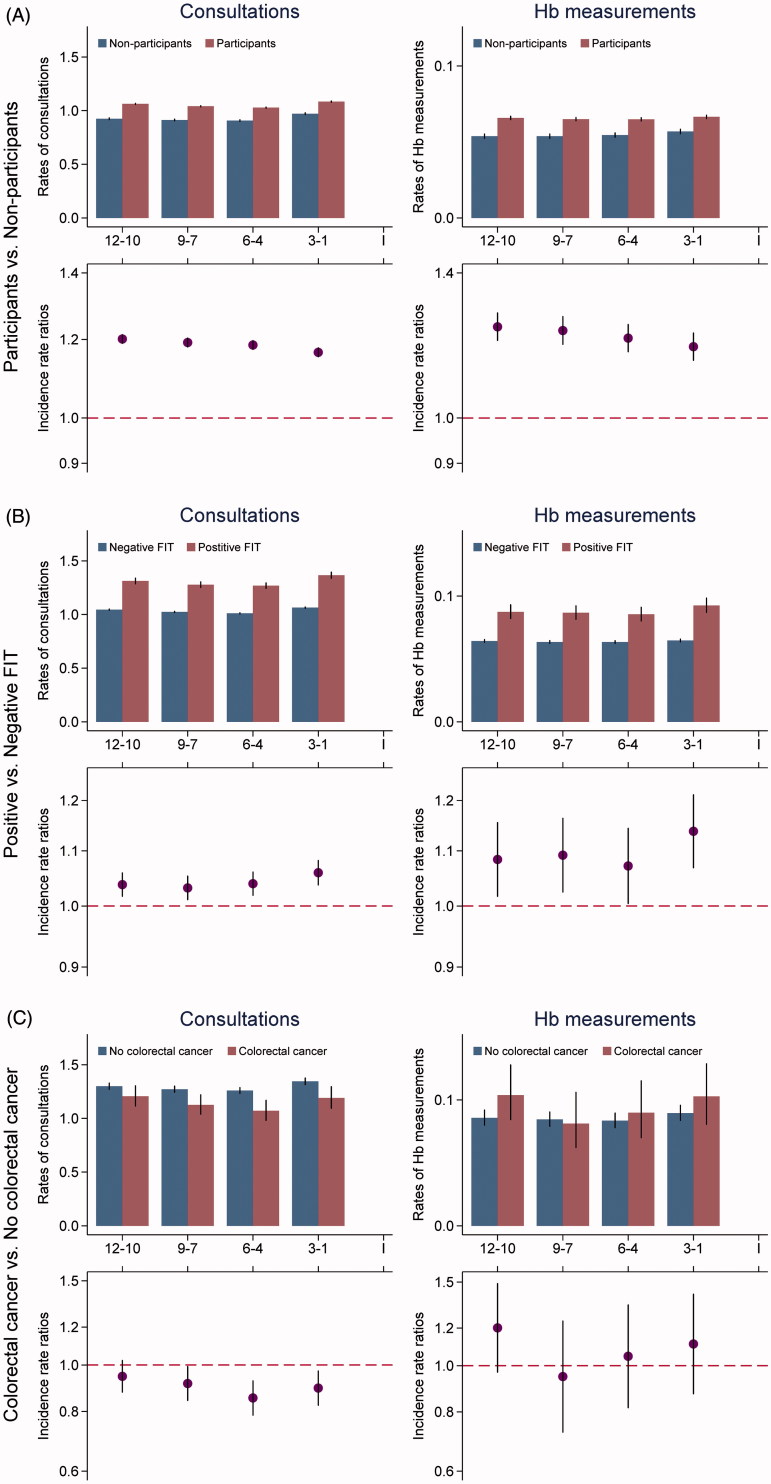
(A-C) Mean rates of daytime face-to-face consultations and haemoglobin measurements for subgroups in the screening for CRC. Estimates are for 3-month intervals, 12 months preceding invitation to screening. The upper graph illustrates the unadjusted average consultation rate for individuals in subgroups. The lower graph illustrates the IRRs for comparison of subgroups adjusted for age, gender, country of origin, educational level, labour market affiliation, marital status, CCI score, prescriptions of medicine against haemorrhoids, NSAIDs, acetylsalicylic acids and anticoagulant drugs.

*Participants vs. non-participants:* Participants in screening were more often females and had higher SES and less comorbidity than non-participants (Table 1). During the year preceding the screening invitation, participants had significantly higher consultation rates and haemoglobin measurements than non-participants (Figure 2(A)). This was also seen when extending the study period to three years (results not shown).

**Table 1.** Characteristics of individuals included in the analyses on diagnostic activity.

	Participants		Non-participants		Positive FIT		Negative FIT		Colorectal cancer		No colorectal cancer	
	*n*	*%*	*n*	*%*	*n*	*%*	*n*	*%*	*n*	*%*	*n*	%
Total	245,299		130,899		16,206		229,093		907		13,955	
Gender†												
Male	113,632	46.3	71,912	54.9	9,083	56.1	104,549	45.6	538	59.3	7,781	55.8
Female	131,663	53.7	58,978	45.1	7,123	43.9	124,540	54.4	369	40.7	6,174	44.2
Age (years)												
50-54	83,138	33.9	54,306	41.5	3,467	21.4	79,671	34.8	87	9.6	3,154	22.6
55-59	35,887	14.6	18,727	14.3	1.910	11.8	33,977	14.8	77	8.5	1,695	12.2
60-64	35,599	14.5	16,003	12.2	2,392	14.7	33.207	14.5	126	13.9	2,102	15.1
65-69	39,734	16.2	15,903	12.2	3,254	20.1	36,480	15.9	198	21.8	2,794	20.0
70-74	50,941	20.8	25,960	19.8	5,183	32.0	45,758	20.0	419	46.2	4,210	30.2
Country of origin												
Danish	229,909	93.7	118,402	90.5	15,360	94.8	214,549	93.7	881	97.1	13,233	94.8
Immigrant (western)	7,569	3.1	5,486	4.2	468	2.9	7,101	3.1	19	2.1	385	2.8
Immigrant (non-western)	7,821	3.2	7,011	5.3	378	2.3	7,443	3.3	7	0.8	337	2.4
Educational level												
Basic	65,204	26.6	46,202	35.3	5,048	31,1	60,156	26.3	295	32.5	4,258	30.5
Medium	121,980	49.7	59,217	45.2	8,273	51.1	113,707	49.6	467	51.5	7,143	51.2
High	58,115	23.7	25,480	19.5	2,885	17.8	55,230	24.1	145	16.0	2,554	18.3
Labour marked affiliation†												
Working	136,066	55.5	66,792	51.0	6,576	40.6	129,490	56.5	310	34.2	5,924	42.5
Unemployed	37,298	15.2	29,619	22.6	2,691	16.6	34,607	15.1	88	9.7	2,324	16.7
Pension	71,931	29.3	34,479	26.4	6,939	42.8	64,992	28.4	509	56.1	5,707	40.9
Marital status†												
Married/cohabiting	184,196	75.1	77,840	59.5	11,526	71.1	172,670	75.4	665	73.3	10,099	72.4
Single/living alone	61,099	24.9	53,050	40.5	4,680	28.9	56,419	24.6	242	26.7	3,856	27.6
Charlson Comorbidity Index												
Low	186,198	75.9	95.442	72.9	10,591	65.4	175,607	76.7	614	67.7	9,272	66.4
Moderate	47,443	19.3	26,000	19.9	4,109	25.3	43,334	18.9	226	24.9	3,477	24.9
Severe	11,658	4.8	9,457	7.2	1,506	9.3	10,152	4.4	67	7.4	1,206	8.6
Prescription for medicine against haemorrhoids												
No	236,184	96.3	127,613	97.5	15,521	95.8	220,663	96.3	870	95.9	13,396	96.0
Yes	9,115	3.7	3,286	2.5	685	4.2	8,430	3.7	37	4.1	559	4.0
Prescription for NSAID												
No	194,067	79.1	105,945	80.9	12,479	77.0	181,588	79.3	743	81.9	10,691	76.6
Yes	51,232	20.9	24,954	19.1	3,727	23.0	47,505	20.7	164	18.1	3,264	23.4
Prescription for acetylsalicylic acids												
No	214,396	87.4	114,097	87.2	12,942	79.9	201,454	87.9	729	80.4	11,214	80.4
Yes	30,903	12.6	16,802	12.8	3,264	20.1	27,639	12.1	178	19.6	2,741	19.6
Prescription for anticoagulant drugs												
No	228,729	93.2	120,686	92,2	13,955	86.1	214,774	93.8	803	88.5	12,091	86.6
Yes	16,570	6.8	10,213	7.8	2,251	13.9	14,319	6.2	104	11.5	1,864	13.4

† Information on gender, labour marked affiliation and marital status were missing for 13 individuals.

*Individuals with positive FIT vs. individuals with negative FIT:* More males than females had a positive FIT in screening. In addition, individuals with a positive FIT had lower SES, higher CCI score and more often received medicine with an anticoagulatory effect (Table 1). Higher consultation rates were seen for individuals with a positive FIT compared to individuals with a negative FIT, but also a small insignificant increase in the last three months preceding the screening invitation compared with the three-month period earlier (Figure 2(B)). The rate of haemoglobin measurements was generally higher for individuals with positive FIT, also with an insignificant increase in the last three months.

*Individuals with colorectal cancer vs. individuals with no colorectal cancer:* Individuals diagnosed with a screen-detected CRC were more often males and of higher age than individuals with no CRC (Table 1). The consultation rates were lower for individuals who were diagnosed with CRC compared to individuals without CRC (Figure 2(C)). This did not change if the rates were stratified for CRC stages and was seen constantly if the study period was extended to three years (data not shown). There were no significant differences in the rates of haemoglobin measurements.

### Cumulative incidence of CRC in the year following the screening invitation

**Figure 3.**
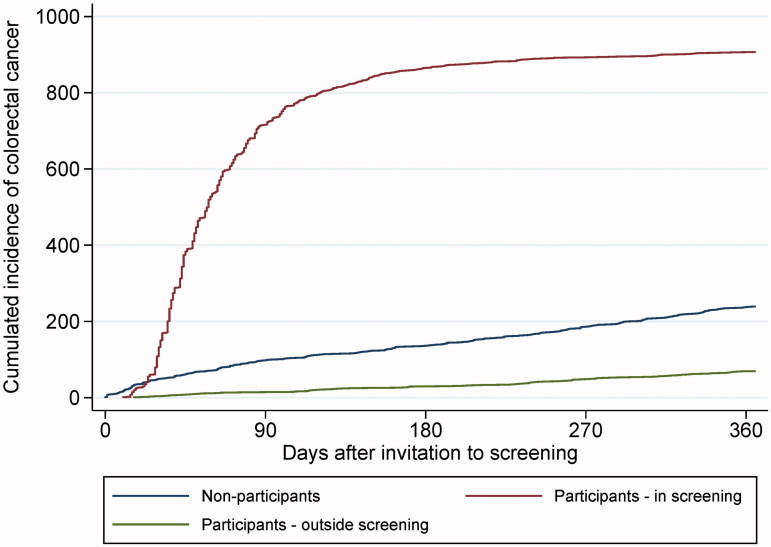
Cumulative incidence of CRC among individuals invited for screening participation, one year following invitation. Incidence was stratified for screening participation. In total, 907 CRCs were diagnosed in the screening (red curve) and 308 CRCs were diagnosed outside the screening (blue and green curves).

One year after the screening invitation, 239 (0.18%) non-participants and 976 (0.40%) participants had been diagnosed with CRC. Among participants, 69 CRC incidents were diagnosed outside the screening programme (Figure 3) implying that in total; 308 (25.3%) of the CRCs diagnosed within the first year after the invitation were identified outside the screening programme.

The characteristics of individuals diagnosed with CRC in the year following the screening invitation are shown in Table 2. Non-participants with CRC were more likely to have low SES and to have higher CCI score compared to participants. The overall risk of being diagnosed with a stage IV CRC was 0.021% (95% confidence interval (95%CI): 0.015; 0.027) for participants and 0.035% (95%CI: 0.026; 0.047) for non-participants, corresponding to a 67% increased risk of late stage (data not shown).

**Table 2.** Characteristics for individuals diagnosed with CRC in the year following the screening invitation.

	Participants diagnosed with CRC in the screening		Participants diagnosed with CRC outside the screening		Non-participants diagnosed with CRC	
	*n*	*%*	*n*	*%*	*n*	*%*
Total	907		69		239	
Gender						
Male	538	59.3	35	50.7	147	61.5
Female	369	40.7	34	49.3	92	38.8
Age (years)						
50-54	87	9.6	10	14.5	28	11.7
55-59	77	8.5	0	–	14	5.9
60-64	126	13.9	9	13.0	47	19.7
65-69	198	21.8	22	31.9	38	15.9
70-74	419	46.2	28	40.6	112	46.8
Country of origin						
Danish	881	97.1	66	95.7	230	96.2
Immigrant (western)	19	2.1	3	4.3	NA	–
Immigrant (non-western)	7	0.8	0	–	NA	–
Educational level						
Basic	295	32.5	24	34.8	84	35.1
Medium	467	51.5	35	50.7	109	45.6
High	145	16.0	10	14.5	46	19.3
Labour market affiliation						
Working	310	34.2	21	36.2	75	31.4
Unemployed	88	9.7	8	13.8	41	17.1
Pension	509	56.1	29	50.0	123	51.5
Marital status						
Married/cohabiting	665	73.3	53	76.8	141	59.0
Single/living alone	242	26.7	16	23.2	98	41.0
Charlson Comorbidity Index						
Low	614	67.7	45	65.2	144	60.3
Moderate	226	24.9	17	24.6	60	25.1
Severe	67	7.4	7	10.1	35	14.6
UICC stage						
I	246	27.1	24	10.0	10	14.5
II	160	17.6	40	16.7	12	17.4
III	171	18.9	51	21.3	13	18.8
IV	51	5.6	46	19.3	13	18.9
Unknown	279	30.8	78	32.7	21	30.4

## Discussion

### Principal findings

Compared to non-participants, screening-participants had higher SES and a higher diagnostic general practice activity in the year preceding the screening. Individuals with a positive FIT had lower SES, more comorbidity and a higher diagnostic activity compared to individuals with a negative FIT. This group also showed a small increase in the three months preceding screening. Individuals diagnosed with CRC in the screening had lower diagnostic activity than individuals with no CRC.

As expected, the screening helped identify cases of CRC. However, approx. 25% of the CRCs diagnosed in the year following the screening invitation were found outside the screening programme. Non-participants with CRC were more likely to have low SES and more comorbidity and had a 67% increased risk of being diagnosed with a stage IV CRC.

### Strengths and weaknesses

A major strength of the study was the large study population. This, together with analysing the diagnostic activity in three-month intervals, ensured a high statistical power for these analyses. However, using three-month intervals might have hidden more abrupt changes in the activity. Therefore, we also performed the analyses with monthly intervals, but this did not change the overall findings and estimates.

Using daytime face-to-face consultations and hemoglobin measurements as proxies for presentation of gastrointestinal symptoms and signs in general practice has both advantages and disadvantages. The approach has previously been used in similar studies and is therefore a well-investigated and accepted method [23,24]. These previous studies have shown that CRC patients visit their GP more than the average population in the time leading up to the diagnosis. Using the Danish National Health Service Register for collecting information on general practice consultations enabled us to leave out consultations with preventive focus as these have specific codes in the register. Therefore consultations in this study can be considered as “new events” of symptoms and disease [12]. However, some contacts may have been due to other symptoms than GI-symptoms.

In general, we consider the internal validity of the study to be high. Information bias was diminished by using Danish registers as the primary data source. These databases contain data collected prospectively and independently of this study. However, the DCCSD is a rather newly established database and a recently published paper has shown a sensitivity for CRC of 72%. Therefore, some screen-detected CRC diagnoses may have been missed and overestimated the amount of CRC found outside screening [15]. Selection bias was minimal due to the random order in which individuals were invited to screening and that all citizens are invited regardless of SES and morbidity. However, we cannot rule out that some selection occurred in relation to who chose to participate in the screening. Furthermore, some of the difference found between subgroups regarding SES and comborbidity may be related to differences in age between the groups. Finally, we cannot rule out the presence of residual confounding from comorbidity and medicine with anticoagulatory effect. Firstly, the CCI score accounts for only 17 comorbid conditions and are based on diagnoses from secondary care. Therefore, it may leave out additional comorbidity that could be a reason for increased health-care seeking in general practice. However, we chose to use the CCI score since it is regarded among the best methods to measure comorbidity and that it was not possible to collect diagnoses registered in primary care since the Danish authorities made the existing database inaccessible for researchers in 2015. Secondly, we did not have the possibility of collecting information on over-the-counter sale of drugs with anticoagulatory effect (primarily NSAIDs) and information on diagnoses from general practice. This residual confounding may therefore account for some of the difference between subgroups.

The reported results on the incidence of CRC outside the screening programme are generalizable to other countries using FIT-based screening, but are dependent on participation rate and the screened age group. Furthermore, the results on diagnostic activity are generalizable to health care settings similar to the Danish.

### Comparison with other research

From the present results it seems that the majority of individuals with a screen-detected CRC are not symptomatic as indicated by similar use of general practice. Thus, it seems unlikely that these individuals may be diagnosed earlier in general practice. However, previous questionnaire studies have indicated that approx. 75-80% of individuals with a positive FIT in a screening programme have lower GI symptoms [4–6]. An explanation for these findings could be recall bias among individuals with a screen-detected CRC. Another explanation could be that the symptoms the patient experience are not serious enough for the patient to consult a GP. Nevertheless, our findings represent a mean for all patients. This could imply that a proportion of the population attend the GP more often before attending the screening due to gastro-intestinal symptoms. However, in this study, we cannot identify who or how many this concerns.

In the analyses comparing the diagnostic activity for participants vs. non-participants, we found that participants in screening generally had higher diagnostic activity in the years preceding screening invitation. This is supported by the literature which shows that low SES and a low use of health care services are known to be related to non-participation in screening [8]. We therefore consider the difference a consequence of another health care seeking behaviour and a lower threshold for acting on symptoms, rather than a true difference in symptom prevalence [25].

In line with the literature, we found that CRC occurs in general practice in the screened age group [7,26]. It has been shown that individuals diagnosed with CRC in general practice attend their GP more in the year preceding the diagnosis than the background population [24]. This may indicate a diagnostic window to detect CRC earlier. Recent studies have suggested that the FIT can be used outside screening in general practice to detect CRC in symptomatic individuals [27–30]. In consideration of this, the FIT may be used in this diagnostic window to reduce the time to diagnosis. In relation to our findings, the majority of screening non-participants were individuals with low SES and high levels of comorbidity. We also saw that this population had higher frequency of positive FITs when they participated in the screening and therefore, the FIT may be a useful diagnostic tool in general practice to detect CRC in this population. However, further research is needed on this topic.

In conclusion, we did not find evidence to support that the majority of individuals with a positive FIT or CRC diagnosed in the screening programme were symptomatic. However, CRC will still occur in general practice in the screened population, and special notice has to be paid on non-participants since they are often deprived and have higher risk of late stage CRC.
